# Post-translational modification of OCT4 in breast cancer tumorigenesis

**DOI:** 10.1038/s41418-018-0079-6

**Published:** 2018-03-06

**Authors:** Yunhee Cho, Hyeok Gu Kang, Seok-Jun Kim, Seul Lee, Sujin Jee, Sung Gwe Ahn, Min Jueng Kang, Joon Seon Song, Joon-Yong Chung, Eugene C. Yi, Kyung-Hee Chun

**Affiliations:** 1 Department of Biochemistry and Molecular Biology, Yonsei University College of Medicine, 50 Yonsei-ro, Seodaemun-gu, Seoul, 03722 Republic of Korea; 2 Brain Korea 21 PLUS Project for Medical Science, Yonsei University College of Medicine, 50 Yonsei-ro, Seodaemun-gu, Seoul, 03722 Republic of Korea; 3 Department of Biomedical Science College of Natural Science, Chosun University, 309 Pilmun-daero, Dong-gu, Gwangju, 61452 Republic of Korea; 4 Department of Surgery, Gangnam Severance Hospital, Yonsei University College of Medicine, 50 Yonsei-ro, Seodaemun-gu, Seoul, 03722 Republic of Korea; 5 Department of Molecular Medicine and Biopharmaceutical Sciences, School of Convergence Science and Technology and College of Medicine or College of Pharmacy, Seoul National University, Seoul, South Korea; 6 Department of Pathology, Asan Medical Center, University of Ulsan College of Medicine, Seoul, 05505 Republic of Korea; 7 Experimental Pathology Laboratory, Laboratory of Pathology, National Cancer Institute, National Institutes of Health, Bethesda, MD 20892 USA

**Keywords:** Oncogenes, Ubiquitin ligases

## Abstract

Recurrence and drug resistance of breast cancer are still the main reasons for breast cancer-associated deaths. Cancer stem cell (CSC) model has been proposed as a hypothesis for the lethality of breast cancer. Molecular mechanisms underlying CSC maintenance are still unclear. In this study, we generated mammospheres derived from breast cancer MDA-MB231 cells and MCF7 cells to enrich CSCs and performed DNA microarray analysis. We found that the expression of carboxy terminus of HSP70-interacting protein (CHIP) E3 ubiquitin ligase was significantly downregulated in breast CSCs. CHIP depletion increased mammosphere formation, whereas CHIP overexpression reversed this effect. We identified interactomes by mass spectrometry and detected CHIP directly interacted with OCT4, a stemness factor. CHIP overexpression decreased OCT4 stability through proteasomal degradation. CHIP induced OCT4 ubiquitination, whereas H260Q, a catalytic CHIP mutant, did not. Interestingly, we determined that OCT4 was ubiquitinated at lysine 284, and CHIP overexpression did not degrade K284R mutant OCT4. CHIP overexpression decreased the proliferation and side population of breast cancer cells, but these were not occurred in K284R mutant OCT4 overexpressed cells. Only 1000 cells showing CHIP depletion or OCT4 overexpression sufficiently generated breast tumors and lung metastases in xenografted mice. Ubiquitination-defective mutant of OCT4(K284R) overexpressed cells drastically generated tumor burdens in mice. Patients with breast cancer who showed low CHIP expression had poor survival probability. Taken together, we suggest that CHIP-induced OCT4 ubiquitination is important in breast CSCs. Regulation of CHIP expression and OCT4 protein stability is a considerable approach for breast cancer therapy.

## Introduction

Breast cancer is the second leading cause of cancer death in women [1]. Therapeutic advancement has substantially improved 5-year survival rate in patients with breast cancer; however, recurrence and subsequent drug resistance are the main cause of breast cancer-related deaths [2]. Breast cancer lethality can be explained using a cancer stem cell (CSC) model, which suggests that malignancies arise from a small subset of CSCs [3]. Aggressive tumors contain higher proportion of CSCs than benign tumors, and tumor malignancy is correlated with CSC proportion. Like stem cell regulatory pathways, Wnt/β-catenin, Notch, Hedgehog, and bone morphogenetic protein pathways play important roles in CSC regulation [4]. Potential CSC markers include cell surface receptors such as CD133, CD44, and EpCAM; enzymes such as aldehyde dehydrogenase; and transcriptional factors such as SRY-box 2 (SOX2), Nanog homeobox (Nanog), and POU class 5 homeobox 1 (POU5F1 or OCT3/4) [5]. However, limited evidence is available on CSCs and on mechanisms underlying CSC maintenance in tumors [6]. Therefore, we characterized breast CSCs by establishing mammosphere cultures. Because CSCs likely are a small population of cells among cancer cells, mammosphere cultures can be established to identify and to enrich CSCs [7]. Dontu et al. showed that serum-free non-adherent cultures enrich breast stem cells within a population of primary human mammary epithelial cells [8]. This technique was used to enrich CSCs from breast cancer cells.

In this study, we generated mammospheres from MCF7 and MDA-MB231 breast cancer cells and performed DNA microarray analysis to identify the regulator of breast cancer CSCs. We assessed the expression levels of E3 ubiquitin ligases to determine the role of protein stability and homeostasis in CSC maintenance. We found that carboxy terminus of HSP70-interacting protein (CHIP), an E3 ubiquitin ligase, was significantly downregulated in mammospheres derived from MCF7 and MDA-MB231 cells. CHIP is involved in cellular processes such as protein trafficking, degradation, signaling, transcription, and apoptosis [9]. CHIP functions as a chaperone-associated E3 ligase for several proteins and regulates various physiological processes, such as cancers, neurological disorders, cardiac diseases, and bone metabolism [10]. The role of CHIP in cancers is controversial because it is suggested to exert both oncogenic and tumor-suppressive effects. In nude mice with breast tumors, tumor growth and metastasis are negatively correlated with CHIP levels [11]. CHIP seems to regulate the levels of several well-known oncogenic proteins such as TRAF2, NF-ƙB, PTK6, and MIF [12–14]. Further, CHIP degrades several critical oncoproteins such as AKT, MYC, and HIF-1α in various cancers [15–17], indicating its tumor-suppressive role. However, several studies have reported the oncogenic role of CHIP. CHIP promotes the ubiquitination and degradation of FoxO1, antagonizes FoxO1-mediated proapoptotic signaling, and enhances survival and proliferation of cells [18]. PTEN is a target of CHIP-mediated ubiquitination and degradation in prostate cancer cells [19]. However, the exact role of CHIP in CSCs is not elucidated, yet.

In this study, we determined mechanisms underlying CSC maintenance in breast tumors. We found that breast CSCs showed downregulation of CHIP expression and regulation of post-translational modification of OCT4, a stemness factor, because OCT4 dosage is important for determining cell fate of embryonic stem cells and cancer tumorigenesis. Moreover, we determined how downregulation of CHIP regulates OCT4 expression in CSCs and in breast tumorigenesis, and whether regulation of OCT4 stability is a reasonable approach for breast cancer therapy.

## Results

### CHIP E3 ligase is downregulated in mammospheres derived from MCF7 and MDA-MB231 breast cancer cells

**Fig. 1:**
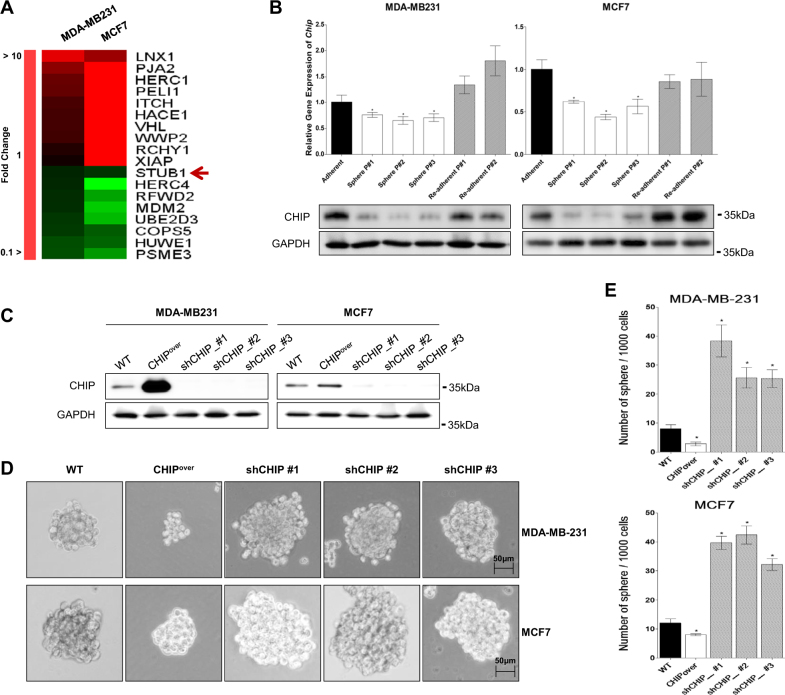
CHIP expression affects mammosphere-forming ability. **a** Expression of different E3 ligases was compared by performing microarray analysis of MDA-MB-231 and MCF7 cells in mammosphere and normal cultures. **b** CHIP expression level was detected by performing qRT-PCR (upper) and western blotting (lower). GAPDH was used as a loading control. **c** The cells were infected with lentiviral vectors to generate the indicated stable cell lines. CHIP overexpression and depletion were confirmed by performing western blotting. **d** Mammosphere-forming ability of the stable cell lines was measured for 15 days under sphere-forming conditions, and (**e**) the number of spheres formed was quantified. Data are presented as mean ± SD (*n = *3). Significant differences are indicated by an asterisk (*p < 0.05); *p* values were calculated using Student’s *t* test

We generated mammospheres from MDA-MB231 and MCF7 cells and determined the alternative expression of E3 ligases by performing DNA microarray analysis (Fig. 1a). We focused on CHIP downregulation. CHIP expression was significantly decreased in serial mammosphere cultures and was restored in re-adherent cultures (Fig. 1b).

Next, we generated stable CHIP-overexpressing cells and three CHIP-depleted cell types (Fig. 1c) and analyzed their mammosphere-forming ability (Fig. 1d,e). CHIP-overexpressing MDA-MB231 or MCF7 cells generated less number of mammospheres that were smaller in size than those generated by parent cells. In contrast, CHIP-depleted MDA-MB231 or MCF7 cells generated four-times higher number of mammosphere that were larger than those generated by parent cells.

### CHIP E3 ligase interacts with OCT4 and induces its proteasomal degradation

**Fig. 2:**
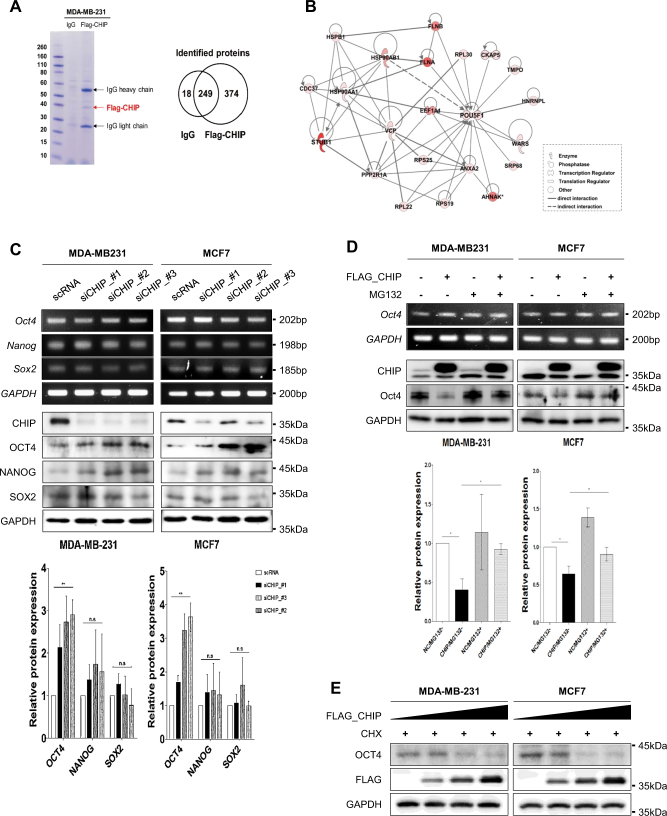
CHIP E3 ligase regulates OCT4 stability through proteasomal degradation. **a** Coomassie blue-stained gel of affinity-purified CHIP complex in MDA-MB-231 cells. The Venn diagram indicates the relationships between proteins identified in each immunoprecipitation complex by performing mass spectrometry. **b** Molecular interaction network between CHIP and OCT4 was determined by performing IPA. **c** MDA-MB-231 and MCF7 cells were transfected with scramble or *CHIP* siRNAs. After 48 h, mRNA (upper) and protein (lower) levels of the indicated genes were detected by performing RT-PCR and western blotting, respectively. **d** The cells were transfected with empty and FLAG–CHIP expression vectors for 40 h and were treated with 20 μM MG132 for 8 h. **e** The cells were transfected with increasing concentrations of the FLAG–CHIP expression vector for 40 h and were treated with 20 μM CHX for 8 h. OCT4 and FLAG–CHIP levels were detected by performing western blotting, with GAPDH as a loading control

We identified CHIP-interacting proteins in FLAG-tagged CHIP-expressing MDA-MB231 cells by mass spectrometry analysis (Fig. 2a). From a total of 641 unique proteins identified, we selected CHIP-interacting proteins with exception of IgG-specific and keratin proteins, and integrated the CHIP-specific proteins into the protein interaction network using the Ingenuity Pathway Analysis (IPA) software. The IPA software generated the development-related proteins that are associated with CHIP (Supplementary Fig. S1). Interestingly, some CHIP-interacting proteins were associated with the self-renewal and stemness of cells, and CHIP appeared to be interacted with OCT4 (Fig. 2b). Firstly, we analyzed the public data (Kaplan–Meier plot analysis) to examine the correlation between OCT4 expression and survival probability (Supplementary Fig. S2). Patients with breast cancer showing high *OCT4* expression showed poor post-progression survival, suggesting that OCT4 expression is a reliable marker for tumor progression and survival of patients with breast cancer.

To determine whether CHIP regulated OCT4 stability, we examined OCT4 expression in the absence of CHIP. Compared with scRNA-transfected cells, OCT4 levels increased in CHIP-depleted cells in an mRNA-independent manner (Fig. 2c). CHIP depletion did not significantly affect the mRNA and protein expression of Nanog and SOX2, suggesting that OCT4 is a direct target of CHIP. We also quantitated the protein changes by CHIP silencing (Fig. 2c below).

Overexpression of CHIP decreased OCT4 protein expression in an mRNA-independent manner, and treatment with proteasome inhibitor MG132 restored OCT4 levels (Fig. 2d). Protein levels of OCT4 reduced in CHIP-overexpressing cells treated with protein synthesis inhibitor cycloheximide (CHX) (Supplementary Fig. S3A), and were correlated with the concentration of CHIP transfected (Fig. 2e). These data suggest that CHIP regulates OCT4 stability through proteasomal degradation.

### CHIP E3 ligase interacts with OCT4 in a chaperon-dependent manner

**Fig. 3:**
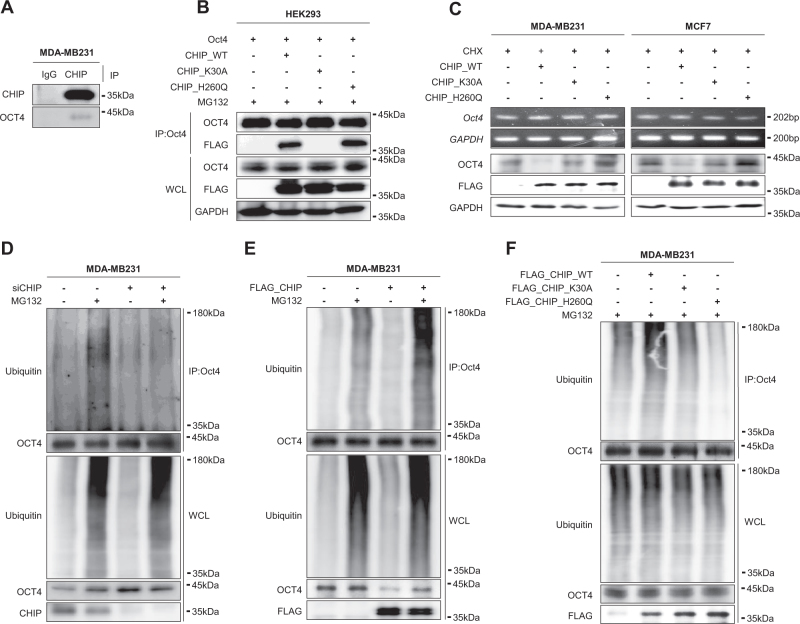
CHIP E3 ligase interacts with OCT4 and induces its polyubiquitination. **a** Interaction between CHIP and OCT4 in MDA-MB-231 cells was detected by performing immunoprecipitation assay. **b** HEK293 cells were transfected with vectors expressing WT FLAG–CHIP or the indicated CHIP mutants K30A and H260Q and were cotransfected with OCT4 expression vector for 40 h and were treated with 20 μM MG132 for 8 h. Interaction of OCT4 with CHIP (WT or mutant) was detected by performing immunoprecipitation assay. **c** The cells were transfected with the indicated vectors for 40 h and were treated with 20 μM CHX for 8 h. GAPDH was used as a loading control. MDA-MB-231 cells were transfected with *CHIP* siRNA (**d**), FLAG–CHIP-overexpressing vector (**e**), and WT or mutant FLAG–CHIP-expressing vector (**f**). After 40 h, the transfected cells were treated with 20 μM MG132 for 8 h. Cell lysates were prepared in denaturing condition and were immunoprecipitated using anti-OCT4 antibody. OCT4 polyubiquitination was detected by performing western blotting

Interaction of CHIP with OCT4 was confirmed in MDA-MB231 cells (Fig. 3a). Notably, OCT4 did not interact with tetratricopeptide repeat (TPR) domain-mutated CHIP (K30A) but interacted with wild-type (WT) CHIP or its E3 ligase functional negative catalytic site mutant H260Q (Fig. 3b). This suggested that the interaction between CHIP and OCT4 was mediated by chaperone proteins because CHIP contains an HSP70 and HSP90 complex-interacting E3 ligase at the TPR domain [20]. We treated MDA-MB 231 and MCF7 cells with an HSP90 inhibitor 17-AAG. 17-AAG treatment significantly decreased OCT4 levels but did not affect CHIP expression (Supplementary Fig. S3B). WT CHIP overexpression decreased OCT4 stability, whereas K30A and H260Q mutant CHIP overexpression did not affect OCT4 stability (Fig. 3c). CHIP depletion restored 17-AAG-induced decrease in OCT4 expression (Supplementary Fig. S3C).

### CHIP E3 ligase induces OCT4 ubiquitination

Overexpression of CHIP E3 ligase functional negative catalytic site mutant H260Q stabilized OCT4 protein expression (Fig. 3c). Next, we performed ubiquitination assay under denaturing condition. CHIP depletion decreased polyubiquitinated OCT4 levels in both MDA-MB231 (Fig. 3d) and MCF7 cells (Suppl.Fig. S4A), whereas CHIP overexpression increased polyubiquitinated OCT4 levels in both MDA-MB231 (Fig. 3e) and MCF7 cells (Suppl.Fig. S4B). Compared to WT CHIP overexpression increased, H260Q mutant CHIP overexpression significantly decreased, and K30A mutant CHIP overexpression decreased polyubiquitinated OCT4 levels in both MDA-MB231 (Fig. 3f) and MCF7 cells (Suppl.Fig. S4C). These data suggest that CHIP induces OCT4 ubiquitination and degradation.

### CHIP E3 ligase ubiquitinates OCT4 at lysine 284

**Fig. 4:**
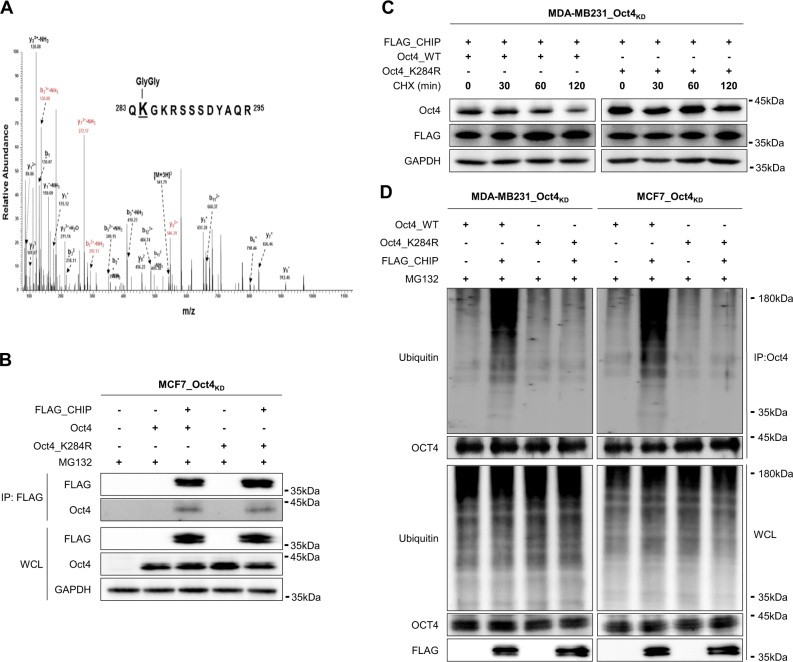
CHIP-induced OCT4 polyubiquitination at K284 regulates OCT4 stability. **a** OCT4 ubiquitination at Lys284, with di-glycine modification in the MS/MS spectrum. **b** MCF7_OCT4_KD_ cells were transfected with the indicated mutant vectors for 40 h, and were treated with 20 μM MG132 for 8 h. Interaction of OCT4 (WT or mutant) with CHIP was detected by performing immunoprecipitation assay. **c** The cells were transfected with increasing concentrations of the FLAG–CHIP-expressing vector and were cotransfected with OCT4_K284R-expressing vector for 40 h, followed by treatment with 20 μM CHX for 8 h. Mutated OCT4 and FLAG–CHIP levels were determined by performing western blotting, with GAPDH as a loading control. **d** The cells were transfected with the indicated vectors for 40 h and were treated with 20 μM MG132 for 8 h. Cell lysates were prepared in denaturing condition and were immunoprecipitated using anti-OCT4 antibody. OCT4 polyubiquitination was determined by performing western blotting

From Mass spectrometric analysis of OCT4, we identified the ubiquitination site at lysine 284 (K284), suggesting that CHIP mediates ubiquitination of OCT4 (Fig. 4a). We further confirmed K284 as the predicted ubiquitination site in OCT4 using BDM-PUB which is a prediction program for potential ubiquitination sites in proteins (Suppl.Fig. S5). To determine whether CHIP decreased OCT4 stability through K284 ubiquitination, we generated endogenous OCT4-depleted stable MDA-MB231 or MCF7 cells (OCT4_KD_ cells) (Supplementary Fig. S6A). We then generated an ubiquitination-defective mutant of OCT4 (OCT4_K284R), and overexpressed WT OCT4 or OCT4_K284R in the endogenous OCT4-depleted cells (OCT4_KD_ cells). As shown in Fig. 4b, the interaction with WT OCT4 or OCT4_K284R and CHIP were detected by immunoprecipitation assay. Compared to OCT4 degradation after co-overexpression of CHIP, overexpression of OCT4_K284R did not show OCT4 degradation by co-overexpression of CHIP in OCT4_KD_ cells (Fig. 4c). Proteosomal degradation of OCT4 was not detected after overexpression of OCT4_K284R in OCT4_KD_ cells (Supplementary Fig. S6B). Moreover, CHIP overexpression did not increase polyubiquitinated OCT4_K284R levels compared to WT-OCT4 levels (Fig. 4d). These data indicate that CHIP polyubiquitinates OCT4 at K284. We also determined in vitro ubiquitination assay with CHIP and mutant OCT4_K284R and CHIP ubiquitinated wild-type OCT4, whereas it could not ubiquitinate mutant OCT4_K284R (Supplementary Fig. S7).

### CHIP E3 ligase overexpression decreases CSC population through OCT4 ubiquitination

**Fig. 5:**
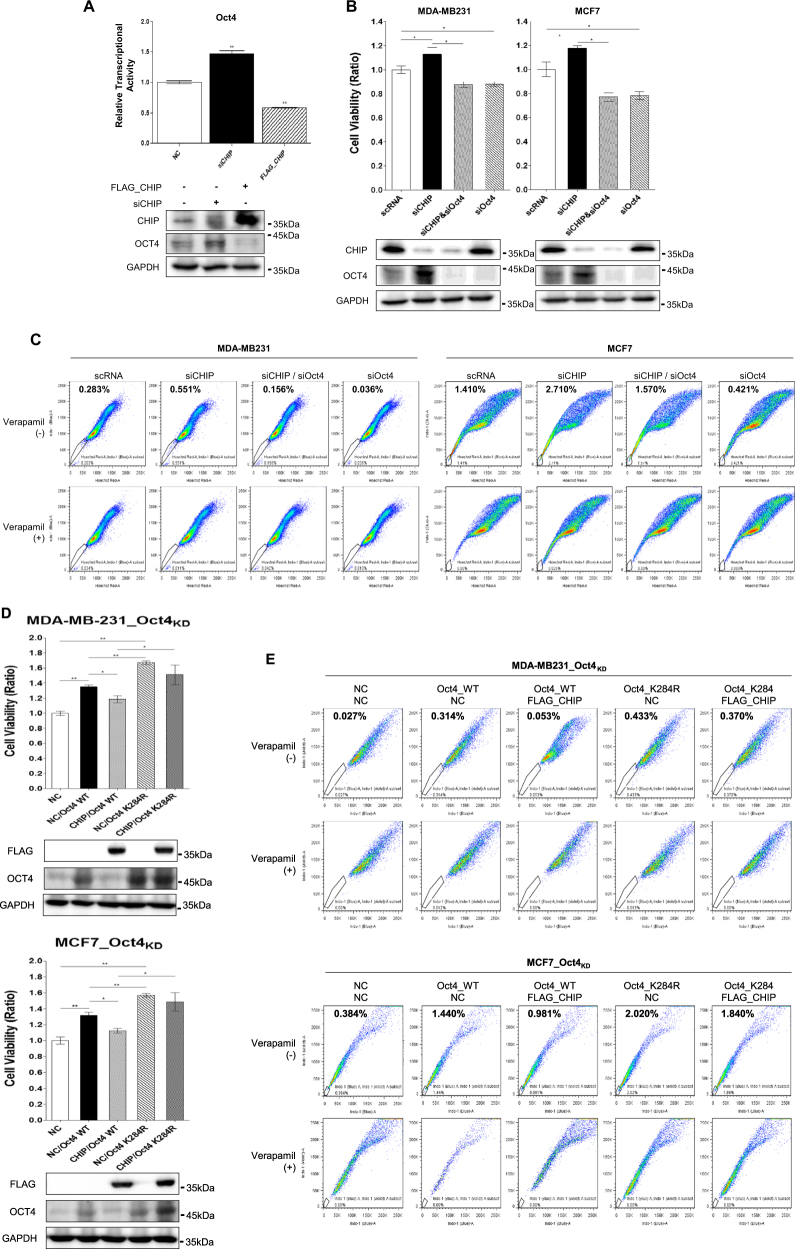
CHIP-induced OCT4 polyubiquitination at K284 regulates cell viability and side population. **a** Transcriptional activation in MCF7 cells was measured by performing the reporter assay. The cells were transfected with OCT4 reporter vector alone or were cotransfected with *CHIP* siRNA or FLAG–CHIP-expressing vector for 48 h. **b**, **c** The cells were transfected with the indicated siRNAs alone or in combination for 48 h, and cell viability was detected by performing WST assay, and side population was analyzed, as described in Materials and methods. **d**, **e** The cells were transfected with the indicated expression vectors alone or in combination for 48 h and cell viability was detected by performing WST assay, and side population was analyzed, as described in Materials and methods. OCT4 and CHIP levels were detected by performing western blotting, with GAPDH as a loading control. Significant differences are indicated by an asterisk (*p < 0.05, **p < 0.01); *p* values were calculated using Student’s *t*test

We measured the transcriptional activity of the *OCT4* promoter to check whether CHIP regulated the transcriptional activity of OCT4. CHIP depletion increased OCT4 transcriptional activity, whereas CHIP overexpression repressed it (Fig. 5a). Furthermore, downstream target genes of OCT4 were increased in CHIP-depleted cells, whereas decreased in CHIP-overexpressed cells (Supplementary Fig. S8). We also found that CHIP depletion increased cell viability, whereas double depletion of CHIP and OCT4 or OCT4 depletion decreased it (Fig. 5b).

Next, we analyzed side population, a cancer stemness characteristic, after CHIP depletion or CHIP/OCT4 double depletion in both MDA-MB231 and MCF7 cells (Fig. 5c). Whereas side population increased after CHIP depletion, CHIP/OCT4 double depletion or OCT4 depletion dramatically decreased side population in both cells.

We also overexpressed WT OCT4 or OCT4_K284R in the endogenous OCT4-depleted cells and measured the viability of these cells (Fig. 5d). It was found that cell viability increased after WT OCT4 overexpression. Moreover, the viability of cells showing OCT4_K284R overexpression was higher than that of cells showing WT OCT4 overexpression. Interestingly, increased viability of WT OCT4-overexpressing cells decreased after additional CHIP overexpression. However, the viability of OCT4_K284R-overexpressing cells was not affected by additional CHIP overexpression.

Next, we measured the side population to determine whether OCT4_K284R affected the stemness of breast CSCs (Fig. 5e). The side population of MDA-MB231 and MCF7 cells increased after OCT4 overexpression, with higher side population being observed after OCT4_K284R overexpression than after WT-OCT4 overexpression. The increase in the side population after WT-OCT4 overexpression decreased after CHIP overexpression. However, OCT4_K284R overexpression-induced increase in the side population was not affected by CHIP overexpression. These data indicate that CHIP induces OCT4 polyubiquitination at K284, which affects the stability and transcriptional activity of OCT4, a major regulator CSCs.

### CHIP E3 ligase reverses OCT4 overexpression-induced increase in tumor burden and metastasis in a breast cancer xenograft mouse model

**Fig. 6:**
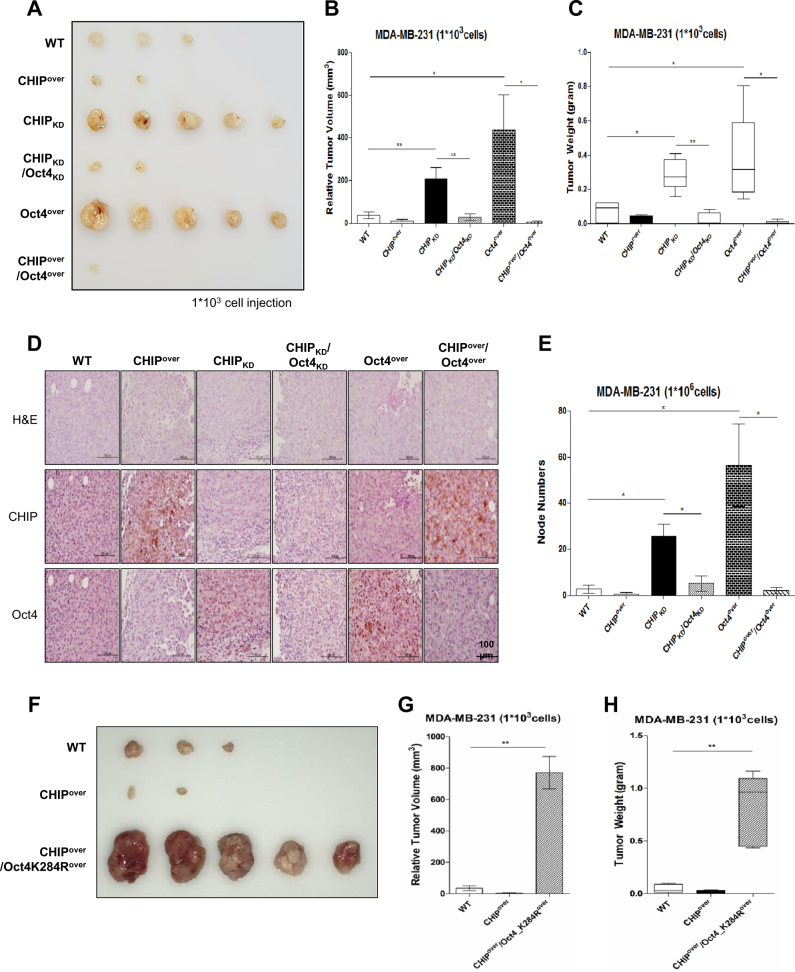
CHIP overexpression decreases tumors and metastatic nodes derived from OCT4-overexpressing cells in vivo. Nude mice were injected with the indicated stable cell lines derived from mammosphere cultured MDA-MB-231 cells (1 × 10^3^) to form subcutaneous xenografts. After 12 weeks, tumors were isolated and were photographed (**a**); their sizes (**b**) and weights (**c**) are presented in a statistical graph. **d** Immunohistochemical analysis of the xenografted tumors by using anti-CHIP and anti-OCT4 antibodies; scale bars, 100 μm. **e** The indicated stable lines derived from MDA-MB-231 cells (1 × 10^6^) were injected into the tail vein of nude mice. After 12 weeks, mouse lungs were isolated and node numbers were counted and presented in a statistical graph. All animal experiments were performed using mammospheres. Nude mice were injected with the indicated stable cell lines derived from mammosphere cultured MDA-MB-231 cells (1 × 10^3^) to form subcutaneous xenografts. After 12 weeks, tumors were isolated and were photographed (**f**); their sizes (**g**) and weights (**h**) are presented in a statistical graph. The data are presented as mean ± SD (*n = *5). Significant differences are indicated by an asterisk (*p < 0.05, **p < 0.01); *p* values were calculated using Student’s *t*test

To examine the in vivo effect of CHIP on breast cancer tumorigenesis, we generated stable MDA-MB231 cells and xenografted 1 × 10^3^ cells cultivated as mammospheres into nude mice. It was displayed by photographs (Fig. 6a) and tumor volume (Fig. 6b) and weight (Fig. 6c). CHIP-overexpressing cells did not produce tumors or produced smaller tumors than those produced by WT cells. In contrast, CHIP-depleted cells produced larger tumors than those produced WT cells. Further, no tumors were produced by CHIP and OCT4 double depleted cells. OCT4-overxpressing cells produced the largest tumors; however, CHIP-overexpression in OCT4-overexpressed cells significantly decreased the size of these tumors.

Immunohistochemical analysis was performed to determine the in vivo expression of CHIP with OCT4 in xenografted tumors (Fig. 6d). We observed that reverse-correlated between CHIP and OCT4; CHIP depletion increased OCT4 expression and CHIP overexpression decreased OCT4 expression in tumor sections. Furthermore, xenografting of cells at different densities produced additional tumors similar to those produced by xenografting 1 × 10^3^ cells (Supplementary Fig. S9, 1 × 10^4^ cells; Supplementary Fig. S10, 1 × 10^5^ cells).

Next, we injected the indicated cells into the tail veins of mice and counted tumors formed in their lungs (Fig. 6e). We observed that CHIP-overexpressing and CHIP/OCT4 double-overexpressing cells rarely produced metastatic tumors in the mouse lungs, whereas CHIP-depleted or OCT4-overexpressing cells frequently produced metastatic tumors in the mouse lungs (Supplementary Fig. S11A and S11B). However, cells showing CHIP/OCT4 double-overexpression rarely produced metastatic tumors. Quantification data for tumor burden and metastasis incidence are presented as a table (Supplementary Fig. S12).

Furthermore, we generated the CHIP and OCT4_K284R overexpressed MDA-MB231 cells and xenografted 1 × 10^3^ cells cultivated as mammospheres into nude mice. It was displayed by photographs (Fig. 6f) and tumor volume (Fig. 6g) and weight (Fig. 6h). Compared to parent cells or CHIP overexpressed cells, ubiquitination mutant OCT4_K284R overexpressing cells generated the largest tumors in xenografted mice, although the cells were co-overexpression of CHIP. These data show that OCT4 overexpression and/or increased its protein stability could increase tumor progression and metastasis in vivo similar to that observed in vitro.

### Patients with breast cancer showing low CHIP E3 ligase expression have poor survival probability

**Fig. 7:**
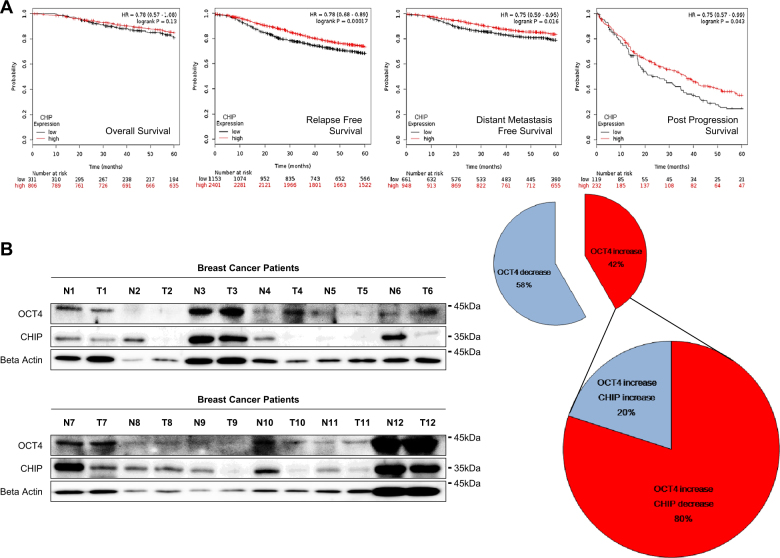
Patients with breast cancer showing low CHIP E3 ligase expression have poor survival probability. **a** Overall, relapse free, distant metastasis and post-progression survival were analyzed with *CHIP* expression level using Kaplan–Meier plot analysis. **b** CHIP and OCT4 expression levels of both malignant (T) and normal (N) tissues were detected using western blotting using western blot. β-Actin was used a normalization control. The data are presented as mean ± SD (*n = *5). We also quantitated the protein changes of CHIP and OCT4, and venn diagram was presented (right). Significant differences are indicated by an asterisk (*p < 0.05, **p < 0.01); *p* values were calculated using Student’s *t* test

We used an online resource and performed Kaplan–Meier plot analysis to explore the survival probability of patients with breast cancer who showed CHIP expression (Fig. 7a). Patients with breast cancer who showed low CHIP expression had poor survival probability, including relapse-free, distant metastasis-free, and post-progression survival. Furthermore, *Oct4* high-expressed breast cancer patients showed the poor post-progression survival probability (Supplementary Fig. S2). Post-progression survival was calculated from tumor progression until death and was correlated with metastasis [21] and the most progressive status [22].

We examined the expressions of CHIP and OCT4 in 12 breast cancer patients by western blotting, respectively. In 10 of 12 breast cancer patients, CHIP expression was downregulated in malignant tissues than in normal counterpart tissues. In contrast, OCT4 expression was higher in malignant tissues compared to their normal counterpart tissues in 5 of 12 breast cancer patients (about 42%, Fig. 7b). In the OCT4 increased patients, CHIP was downregulated in malignant tissues than in normal counterpart tissues, about 80%. Reverse expression of those expressions was also found in five breast cancer patient tissues. However, we could not statistical analysis for the correlation between CHIP and OCT4 because of shortage of sample numbers.

## Discussion

In this study, we established mammospheres from MDA-MB231 and MCF7 breast cancer cells to characterize CSCs. Because CSCs likely are a minority cell population among total cancer cells, their identification and enrichment are a significant challenge [7]. This indicates that stem cells from breast tissue can be performed by the simple mammosphere cultures that enrich highly tumorigenic CSCs, which are probably the most dangerous cells within tumors compared with the bulk population of tumor cells, by using a simple in vitro culture technique. We observed that CHIP E3 ubiquitin ligase was significantly downregulated in mammospheres derived from MDA-MB231 and MCF7 cells compared with that in monolayer cultured cells. We found that CHIP directly interacted with OCT4 and decreased its stability and breast CSC properties. OCT4, a transcription factor encoded by *POU5F1*, belongs to POU family of DNA-binding proteins [23]. These proteins regulate target gene expression by binding to octamer motif ATGCAAAT within promoters or enhancers. OCT4, whose expression is associated with pluripotent properties of stem cells, is an essential factor that controls the early stages of mammalian embryogenesis. *Oct4* mRNA expression level in mouse embryonic stem cells is critical for the maintenance of pluripotency and differentiation toward trophoblast or primitive endodermal and mesodermal lineages. Post-translational modification of OCT4 is important to regulate its function and to cure diseases. Protein kinase A and/or MAPK phosphorylate OCT4 at highly conserved Ser229 (murine) or Ser236 (human) within the POU DNA-binding homeodomain [24]. Phosphorylation at this serine residue sterically hinders both DNA binding and homodimer assembly. ERK1/2 phosphorylates OCT4 at Ser111 to regulate its subcellular distribution and degradation [25]. Small ubiquitin-related modifier (SUMO)-1 targets OCT4 at Lys118 in mice [26]. Sumoylation of OCT4, which does not alter its subnuclear localization, enhances its stability, DNA binding, and transactivation functions. E3 ligases interact directly with OCT4 to promote ubiquitin transfer. OCT4 ubiquitination promotes its degradation and dramatically decreases its transcriptional activity [27–29]. WWP2, Itch, and DPF2 are E3 ubiquitin ligases that specifically interact with OCT4. However, OCT4 site ubiquitinated by these ligases is unclear, yet.

The present study is the first to show that CHIP ubiquitinates OCT4 at Lys284. CHIP overexpression did not degrade mutant OCT4_K284R. Mutant OCT4_K284R overexpression increased the proliferation and side population of breast cancer cells. Excitingly, 1 × 10^3^ of mutant OCT4_K284R overexpressed breast cancer cells drastically generated tumor burdens in xenografted mice. These data strongly indicate that CHIP expression decreases in CSCs and that OCT4 dosage is critical for CSC maintenance. In previous study, it was reported that other E3 ligase WWP and Itch regulates OCT4 stability [27, 28]. However, E3 ligases WWP2 and Itch may not maintain OCT4 stability in breast CSCs because expression of these E3 ligases is upregulated like that of OCT4 in mammosphere cultures. We believe that different E3 ligases regulate OCT4 stability differently in ES cells and CSCs. Therefore, we hypothesize that differential regulation of E3 ligases is critical for CSC survival and maintenance in tumors and needs to be examined further.

Regulation of CHIP in CSCs is unclear. Although we did not elucidate this in the present study, we have discussed it. Limited evidence is available on the regulation of *CHIP* mRNA expression and post-translational modification under different physiological and pathological contexts. *CHIP* mRNA expression is upregulated under various stress conditions such as heat shock and oxidative damage [30, 31]. Stress-induced transcriptional regulation is important under various physiological conditions such as neurodegenerative disorders and heart diseases. TLR2 activation enhances CHIP expression and activity through JNK signaling [32]. Downregulation of *CHIP* mRNA and protein expression has been reported in malignant tissues compared with that in their normal counterparts, including the stomach [33], pancreas [34], and breast [11]. In MC3T3-E1 cells, miR-764-5p inhibits *CHIP* mRNA translation by binding at its 3′-UTR [35]. CHIP is downregulated in osteoblast progenitor cells during osteoblast differentiation. CHIP activity and stability are also regulated through post-translational modification. The N- and C-terminal regions of CHIP are proposed to contain functional phosphorylation sites, which should be confirmed in future studies. Association between CHIP and kinases such as ERK5 and Lim kinase 1 has been reported [36, 37]. ERK5 activation increases CHIP ubiquitin ligase activity possibly through a conformational change in CHIP. Phosphorylation-dependent regulation of CHIP activity is an interesting possibility that needs further investigation. Moreover, mechanisms underlying decreased CHIP expression in CSCs and association between low CHIP expression and CSC survival should be investigated. At present, we are examining the regulation of CHIP expression and activation in CSCs.

Thus, our results indicate that CSCs show decreased CHIP expression and increased OCT4 stability through post-translational modifications to maintain their population and survival during breast cancer progression. Our results also suggest that targeting OCT4 post-translational modification is an ideal approach for breast cancer therapy.

## Materials and Methods

### Cell culture and transfection

The human breast cancer cell lines MDA-MB-231 and MCF7 were obtained from ATCC and were maintained in Dulbecco’s modified Eagle’s medium containing 10% fetal bovine serum (FBS) and 1% antibiotics (Invitrogen) at 37 °C in an atmosphere of 5% CO_2_. Transfection with CHIP, WT and mutant, expression vectors [38] and Oct4 expression vector [39] as well as with CHIP and Oct4 siRNA were performed with Lipofectamine 200 and Lipofectamine RNAiMAX reagent (Invitrogen), according to the manufacturer’s instruction. *CHIP* siRNA #1 (5′-CCCAAGUUCUGCUGUUGGACU-3′), *CHIP* siRNA #2 (5′-GAAGAGGAAGAAGCGAGACAU-3′), *CHIP* siRNA #3 (5′- GCAGUCUGUGAAGGCGCACUU-3′), and *OCT4* siRNA (5′-UUAAGUUCUUCAUUCACUAAG-3′) were purchased from COSMOGENETECH. Cells were collected 2 days after the transfection for use in subsequent experiments.

### Mammosphere culture

Cells (density, 1000 cells per ml) were grown in ultra-low attachment plates (Corning) containing mammary epithelium basal medium (Lonza) supplemented with B27 (Gibco), 20 ng/ml EGF, and 20 ng/ml bFGF (PeproTech). After culturing for 15 days, mammospheres with diameters of >50 μm were counted.

### Mutagenesis and generation of stable cell lines

Point mutation of lysine to arginine at position 284 (K284R) in OCT4 was induced by performing site-directed mutagenesis. Primers used are shown in Supplementary Figure S11. ShRNA-expressing lentiviral vectors for CHIP and OCT4 depletion (CHIP_KD_ and OCT4_KD_ cells) that targeted the 3ʹ-UTR of the encoding genes were purchased from Sigma (*CHIP*: TRCN0000007525, TRCN0000007526, and TRCN0000007527; *OCT4*: TRCN0000235522). Lentivirus production and stable cell line generation were performed, as described previously [40].

### Total RNA isolation and reverse transcription-polymerase chain reaction

RNA was isolated using TRIzol^®^ reagent (Invitrogen), according to the manufacturer’s instructions. Reverse transcription-polymerase chain reaction (RT-PCR) was performed using a reverse transcription system (TOYOBO) and primers listed in Supplementary Figure S13. PCR was performed using instructions given in Ex-Taq (TaKaRa) manual. qRT-PCR was performed using a SYBR Premix (TaKaRa) and AB StepOnePlus Real-Time PCR System, according to the manufacturer’s instructions.

### Microarray analysis

Gene expression in the examined cell lines was analyzed using high-density oligonucleotide microarrays containing 20,889 transcripts (HG-U133 Plus 2.0; Affymetrix). Target preparation and microarray processing were performed as described in Affymetrix GeneChip expression analysis manual. GeneChip analysis was performed using Affymetrix GeneChip manual with Microarray Analysis Suite 5.0, Data Mining Tool 2.0, and Microarray Database software.

### Luciferase assay

OCT4 reporter assay was performed using *OCT4* promoter construct phOCT4-Luc [41]. MCF7 cells were transfected with the CHIP expression vector or *CHIP* siRNA, and β-galactosidase expression vector was used for normalization. After 48 h, luciferase activity was measured using a luciferase assay system (Promega), according to the manufacturer’s instructions.

### Side population analysis

Cells were transfected with the indicated vectors or siRNAs and were harvested after 48 h. Next, 1 × 10^6^ cells were incubated in 1 ml suspension medium (HBSS, 2% FBS, and 10 mM HEPES) containing 5 μg/ml Hoechst 33342 dye (Thermo Fisher Scientific) and 50 μM verapamil (Sigma) at 37 °C for 60 min. The cells were then washed three times with a cold suspension medium and were treated with 2 μg/ml PI solution. A minimum of 20,000 events/sample were collected using FACSDiva and Cell Quest applications (BD Biosciences).

### Cell viability analysis

Cells were grown in 96-well culture plates and were transfected with *CHIP* and *OCT4* siRNAs or CHIP and WT OCT4 and OCT4_K284 expression vectors. After 48 h, WST solution (Daeil) was added to each well. After 1–3 h of incubation, absorbance was measured using ELISA reader at a test wavelength of 450 nm.

### Recombinant protein production and GST-pulldown assay

GST-CHIP, GST-OCT4_WT, and GST-OCT4_K284R were expressed in BL21 cells and purified through a GST-pulldown assay using glutathione Sepharose 4B (GE Healthcare). Glutathione Sepharose 4B was added to the lysates, followed by incubation for 1 h. The samples were washed with PBS and eluted in 10 mM reduced glutathione.

### Ubiquitination assay and in vitro ubiquitination assay

Ubiquitination assay was performed under denaturing condition, as described previously [38, 42].

Recombinant proteins were incubated in 40 mM Tris-HCl (pH7.6), 50 mM NaCl, and 1 mM dithiothreitol with 100 ng of an E1 (UBE1; Boston Biochem), 250 ng of an E2 (UbcH5c; Boston Biochem), 5 μg of ubiquitin (sigma), and 2 mM ATP (Fermentas) for 3 h. The samples were boiled in 2 × SDS buffer, OCT4 and CHIP were detected by western blotting.

### Tissues

Tissue samples were obtained at biopsy from 12 breast cancer patients and their normal counterparts from Gangnam Severance Hospital in Korea. The protocol for the study was approved by the institutional review board (IRB) of the Yonsei University College of Medicine (IRB number: 3–2014–0239). Immediately after biopsy, the tissue samples were frozen in liquid nitrogen and stored at −70 °C until use.

### Immunoprecipitation and western blotting

Cell lysates were incubated with the following antibodies: mouse IgG (sc-3877; Santa Cruz Biotechnology), anti-FLAG antibody (F1804; Sigma), and anti-OCT4 antibody (sc-5279; Santa Cruz Biotechnology). Immunoprecipitation was performed as described previously [39].

Western blotting was performed using the following antibodies: anti-GAPDH (sc-25778), anti-CHIP (sc-66830), anti-Nanog (sc-33759), anti-SOX2 (sc-20088), and anti-OCT4 antibodies (all purchased from Santa Cruz Biotechnology); anti-FLAG antibody (Sigma); and HRP-conjugated anti-Ub antibody (BML-PW0150; ENZO). Proteins of interest were detected using an ECL solution (Amersham Life Science) with LAS-3000 detector (Fujifilm), according to the manufacturer’s directions.

### Immunohistochemistry

Xenografted tumors were obtained and fixed in 4% paraformaldehyde (BIOSESANG). The fixed tumors were embedded in paraffin blocks and were sliced into 0.4-μm-thick sections. CHIP and OCT4 levels in the xenografted tumors were immunohistochemically detected using Vectastain ABC kit and DAB substrate kit (Vector Laboratories).

### Mass spectrometry and network analysis

For identification of CHIP-interacting proteins in MDA-MB231, CHIP immunoprecipitation eluates were separated by SDS-PAGE and subjected to in-gel tryptic digestion following the general protocol [43]. Extracted peptides were suspended in 0.1% FA in water, loaded onto an EASY-Spray C18 column (75 µm × 50 cm, 2 µm) and separated with a 2–35% gradient of 0.1% FA in ACN for 65 min at a flow rate of 300 nL/min. MS spectra were recorded on a Q-Exactive hybrid quadrupole-Orbitrap mass spectrometer (Thermo Fisher Scientific) interfaced with a nano-ultra-HPLC system (Easy-nLC1000; Thermo Scientific). Collected MS/MS raw files were converted to mzXML files using the Trans-Proteomic Pipeline (version 4.4) and analyzed using the Sequest (version 27) algorithm in the SORCERER (Sage-N Research, Milpitas) platform. Protein database search was performed using the Uniprot human database (version 2016.06, 313072 entries). Full tryptic specificity and up to two missed cleavage sites were allowed. Mass tolerances for precursor ions and fragment ions were set to 10 p.p.m. and 1 Da, respectively. Fixed modification for carbamidomethyl-cysteine ( + 57.0215 Da) and variable modifications for methionine oxidation (+15.9949 Da) were used. All proteins with a ProteinProphet probability of ≥95% with minimum two peptides and a PeptideProphet probability of ≥90%, peptide FDR ≤0.3% were identified using Scaffold (version 4.3.2; Proteome Software, Portland, OR, USA).

For ubiquitination site mapping of OCT4, purified OCT4 protein from HEK293 cell line was separated by SDS-PAGE and subjected to in-gel digestion with trypsin/LysC mix (Promega). Collected MS/MS raw files were analyzed using the Proteome Discoverer (version 1.4). Di-glycine modification (GG, +114.043 Da) on the lysine and missed tryptic cleavage at the modified site were used. Human database and other search parameter were set with the same values as above.

Network analysis of selected CHIP-interacting proteins was performed by using the Ingenuity Pathway Analysis (IPA) software (Ingenuity System Inc, USA). Protein interaction networks functionally associated with OCT4 and CHIP were merged to generate a protein interaction network constituted by OCT4 and CHIP.

### Animal experiments

All animal experiments were approved by the Institutional Review Board of the Yonsei University College of Medicine and were performed in specific pathogen-free facilities according to the university’s guidelines for the Care and Use of Laboratory Animals (2015–0376). Xenografted mice and mice showing lung metastasis were generated using the cells from mammosphere culture as described previously [44, 45].

### Kaplan–Meier analysis

Kaplan–Meier analysis of survival curve was performed using http://kmplot.com/analysis and two gene symbols *STUB1* (Affy ID: 217934_x_at) and *POU5F1* (Affy ID: 208286_x_at) for patients with breast cancer.

### Statistical analysis

Two tumors were isolated per mouse and were analyzed to determine mean tumor volume per mouse. Unpaired *t* tests were used to analyze mean tumor volume in the xenografted mice. Statistical analyses were performed using Student’s *t* test with GraphPad Prism software (version 6; GraphPad Software Inc., La Jolla, CA). The data were considered statistically significant at *p* < 0.05.

## Electronic supplementary material

supplementary figure S1

supplementary figure S2

supplementary figure S3

supplementary figure S4

supplementary figure S5

supplementary figure S6

supplementary figure S7

supplementary figure S8

supplementary figure S9

supplementary figure S10

supplementary figure S11

supplementary figure S11

supplementary figure S12

supplementary figure S13
