# Supplementary figures and images for: Post-translational modification of OCT4 in breast cancer tumorigenesis
Source: Cell Death Differ. 2018 Mar 6;25(10):1781–95. doi: 10.1038/s41418-018-0079-6 (PMC6180041; doi:10.1038/s41418-018-0079-6)

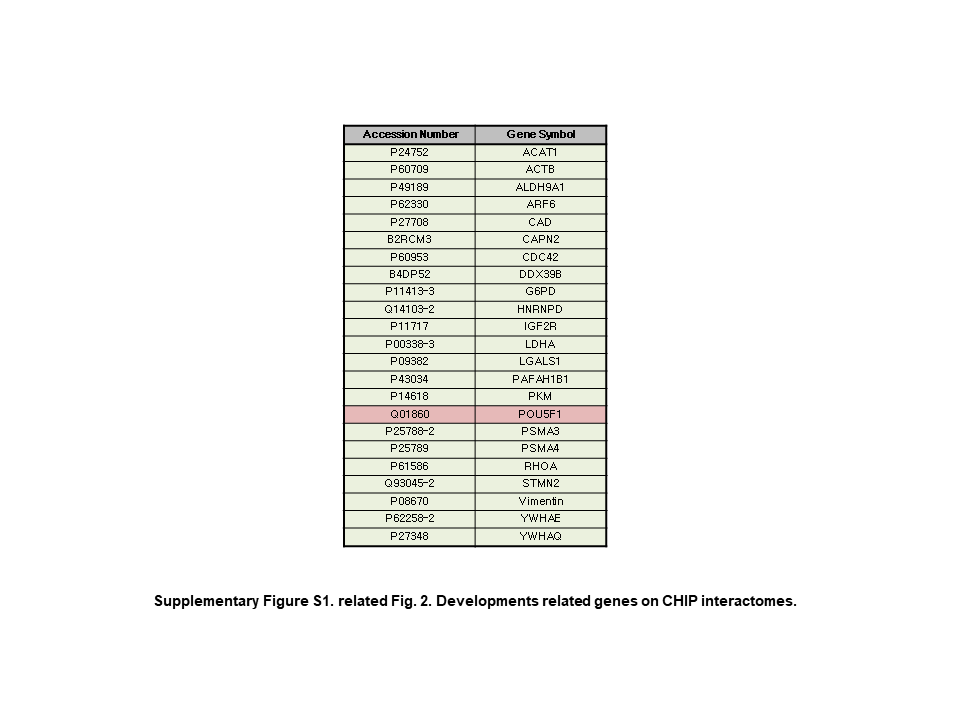

Supplement: Supplementary file 1 — supplementary figure S1 [file 41418_2018_79_MOESM1_ESM.tif]

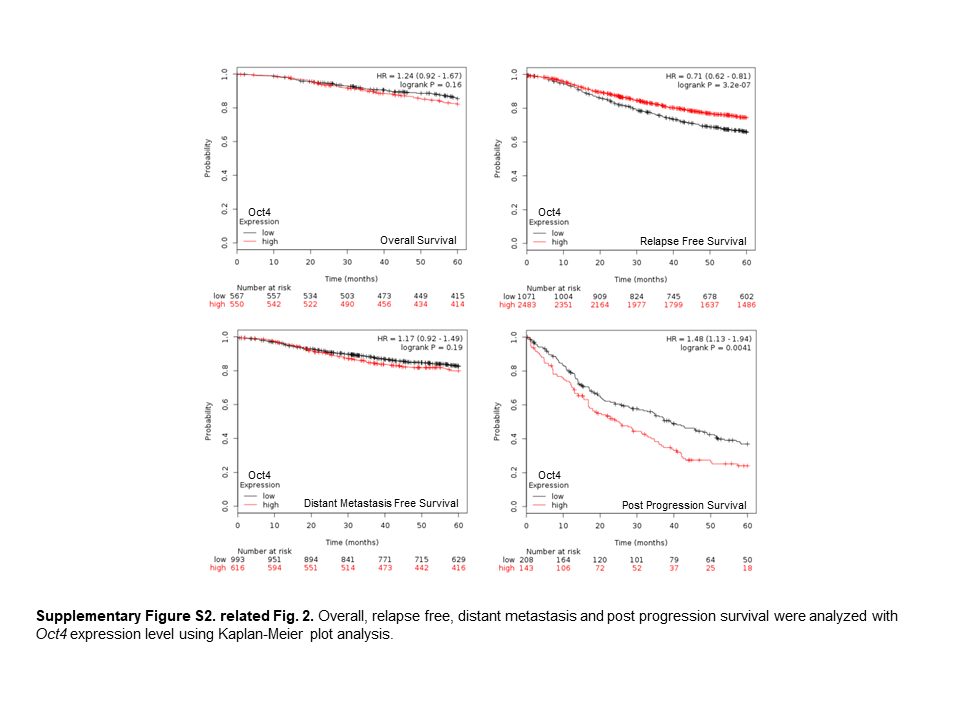

Supplement: Supplementary file 2 — supplementary figure S2 [file 41418_2018_79_MOESM2_ESM.tif]

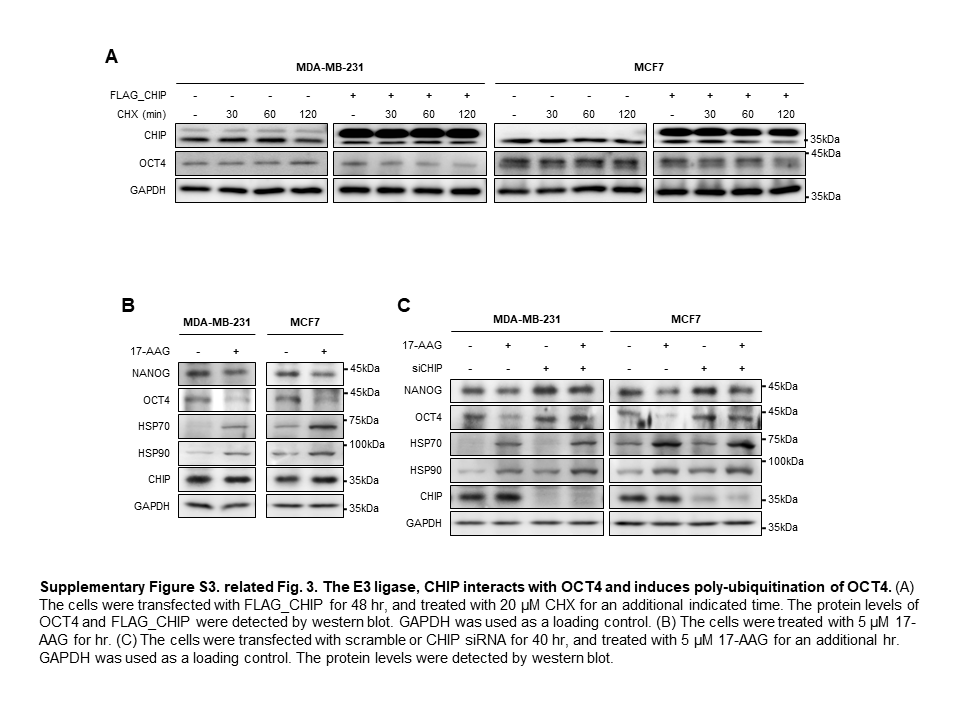

Supplement: Supplementary file 3 — supplementary figure S3 [file 41418_2018_79_MOESM3_ESM.tif]

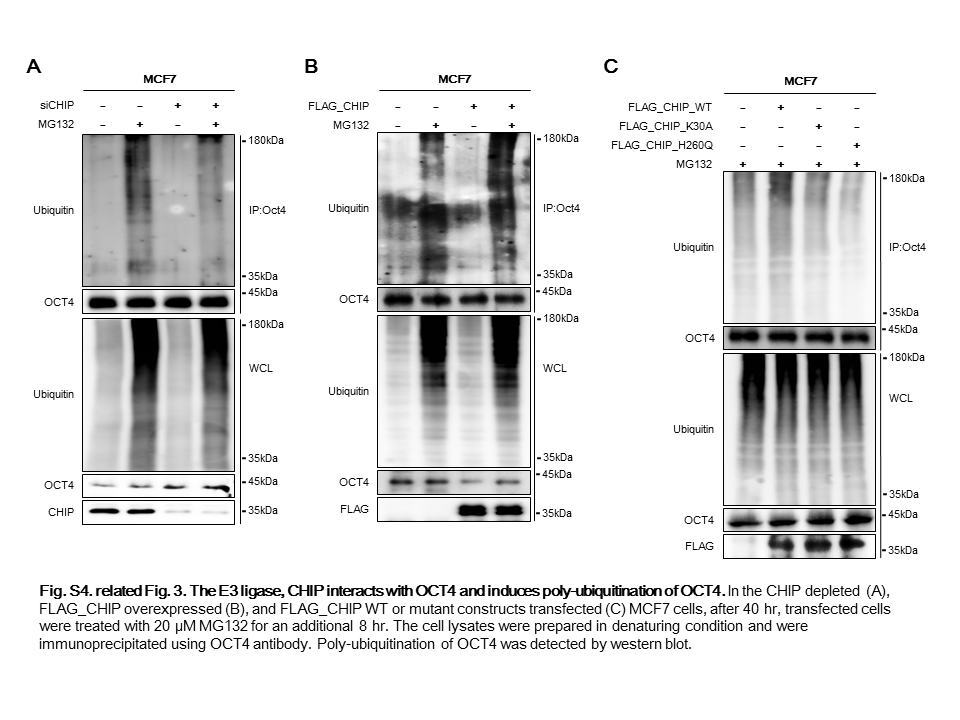

Supplement: Supplementary file 4 — supplementary figure S4 [file 41418_2018_79_MOESM4_ESM.tif]

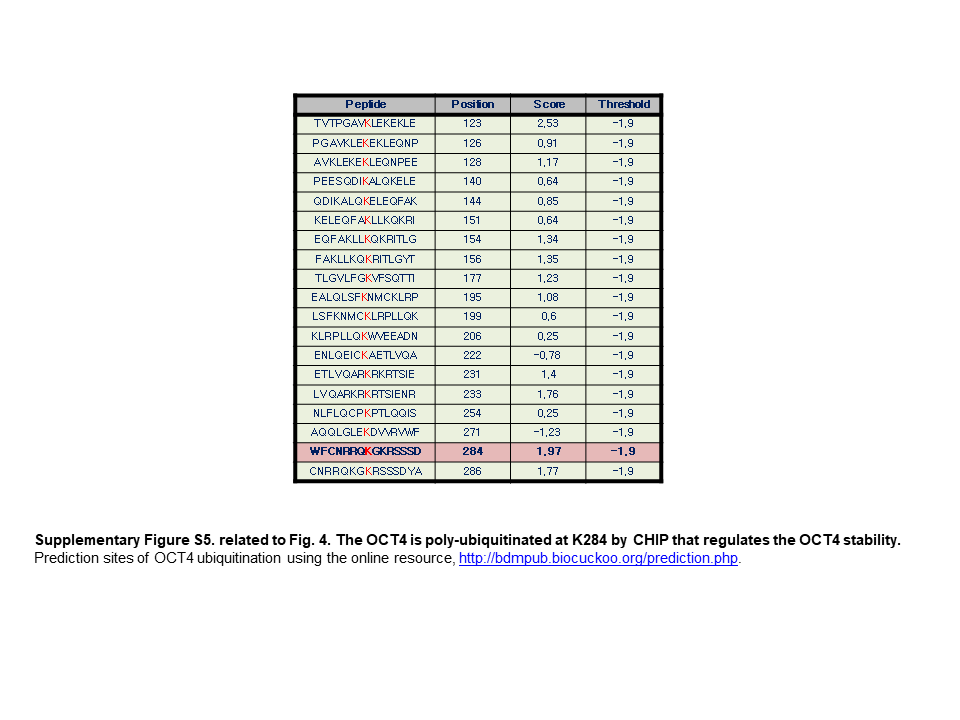

Supplement: Supplementary file 5 — supplementary figure S5 [file 41418_2018_79_MOESM5_ESM.tif]

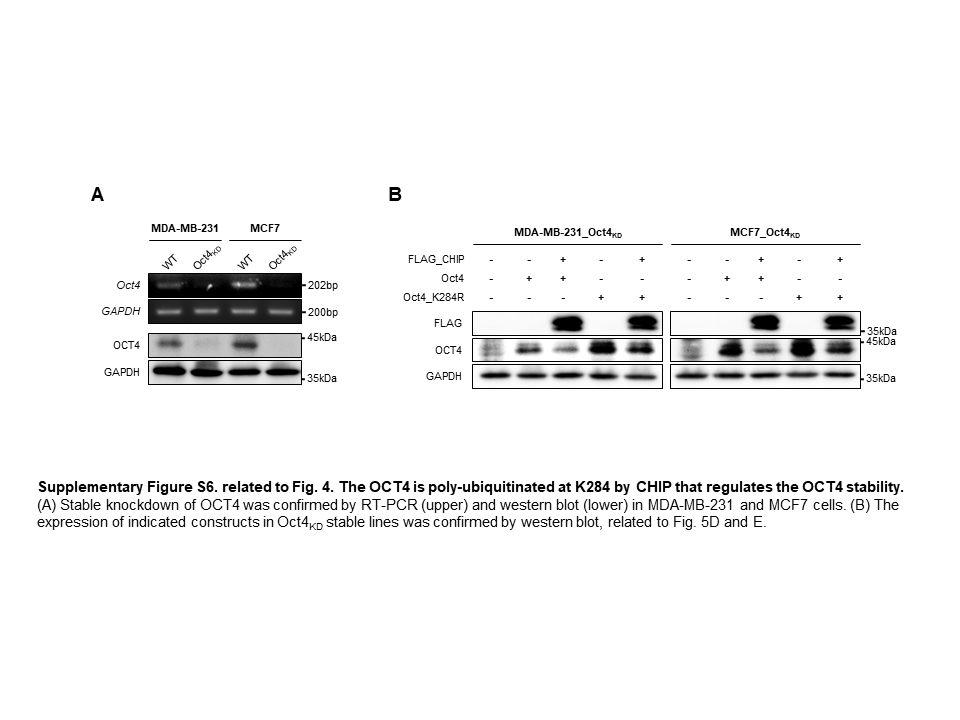

Supplement: Supplementary file 6 — supplementary figure S6 [file 41418_2018_79_MOESM6_ESM.tif]

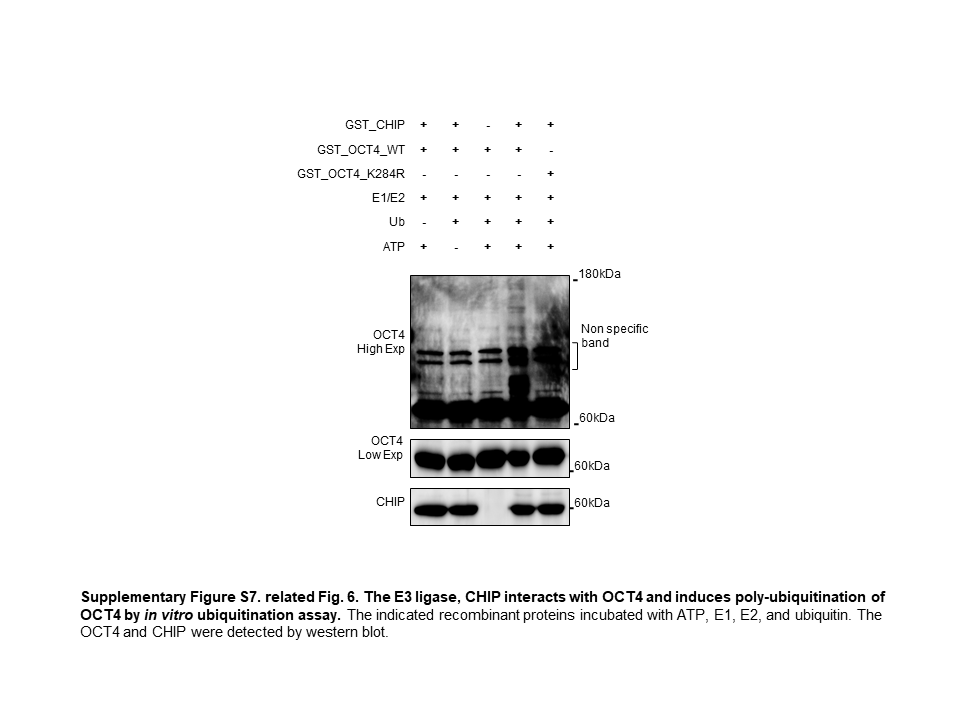

Supplement: Supplementary file 7 — supplementary figure S7 [file 41418_2018_79_MOESM7_ESM.tif]

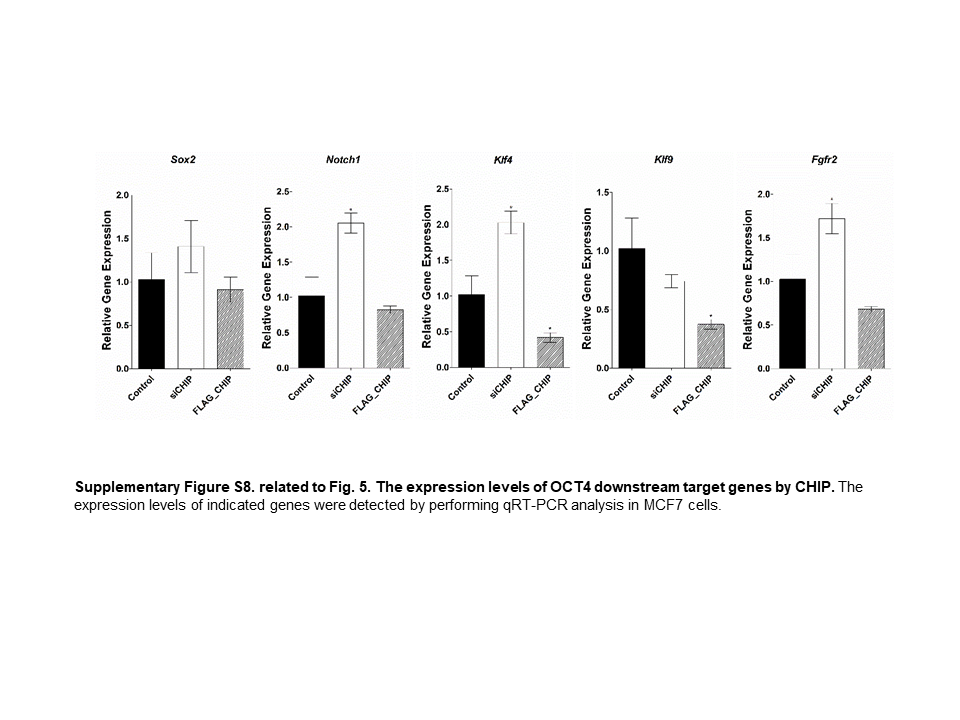

Supplement: Supplementary file 8 — supplementary figure S8 [file 41418_2018_79_MOESM8_ESM.tif]

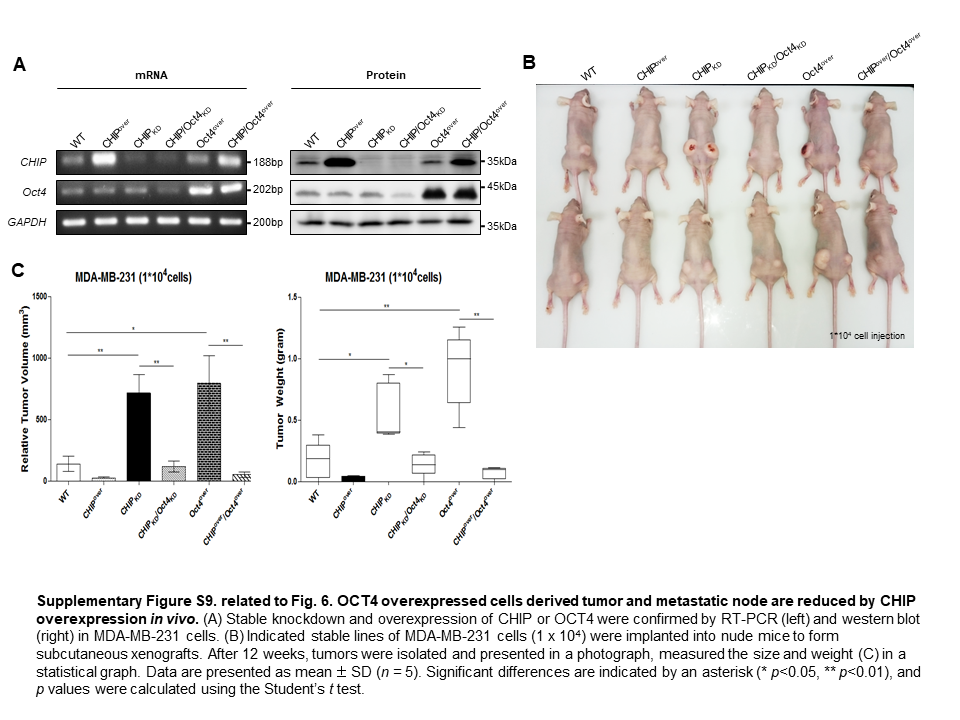

Supplement: Supplementary file 9 — supplementary figure S9 [file 41418_2018_79_MOESM9_ESM.tif]

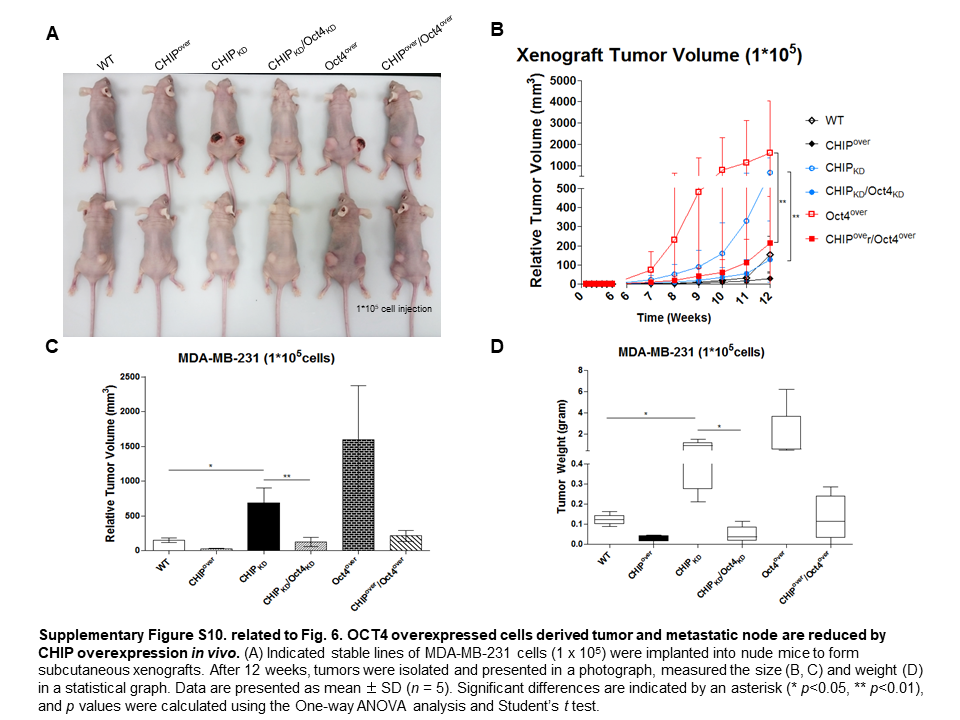

Supplement: Supplementary file 10 — supplementary figure S10 [file 41418_2018_79_MOESM10_ESM.tif]

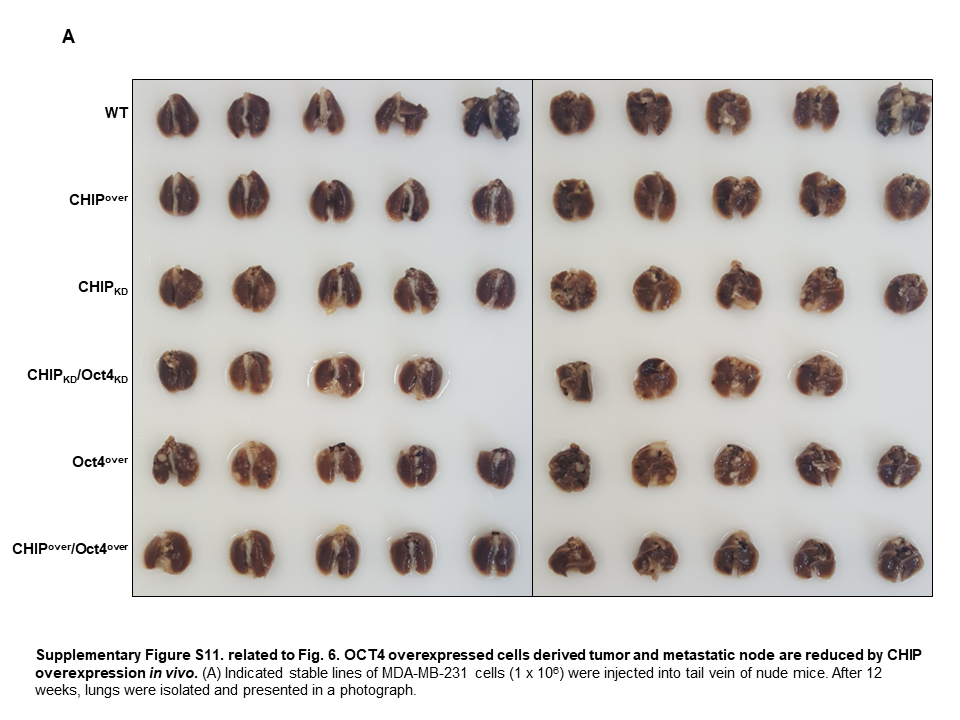

Supplement: Supplementary file 11 — supplementary figure S11 [file 41418_2018_79_MOESM11_ESM.tif]

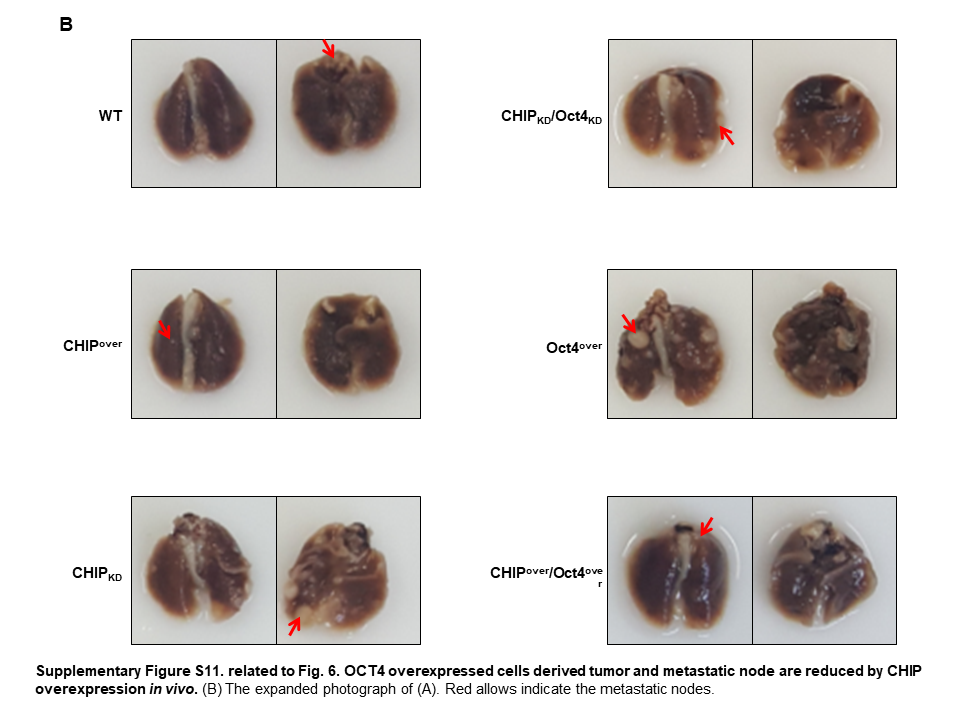

Supplement: Supplementary file 12 — supplementary figure S11 [file 41418_2018_79_MOESM12_ESM.tif]

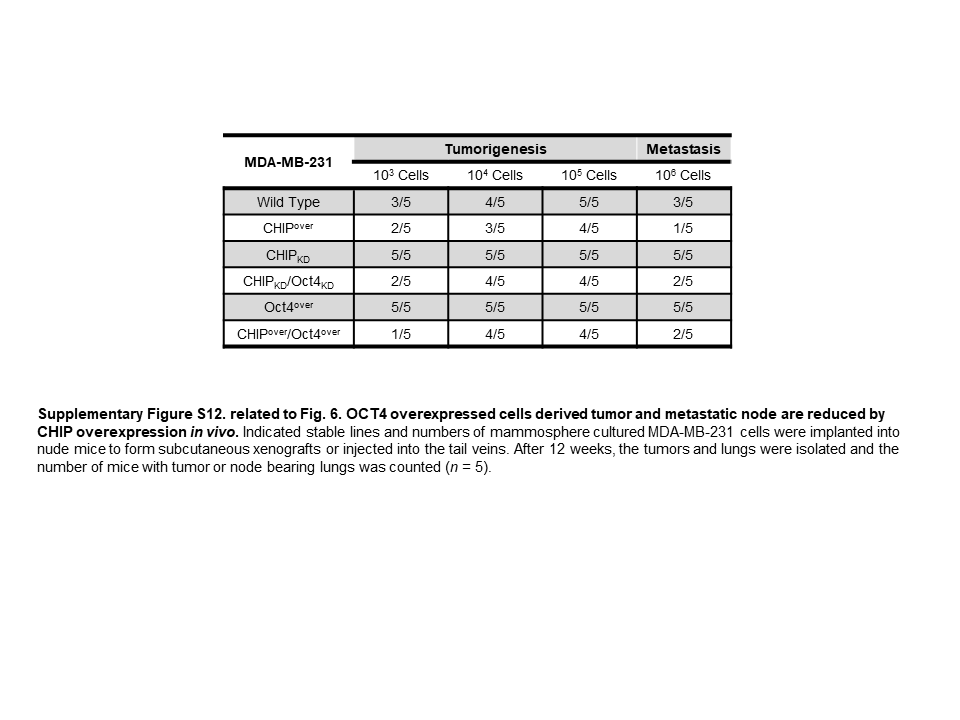

Supplement: Supplementary file 13 — supplementary figure S12 [file 41418_2018_79_MOESM13_ESM.tif]

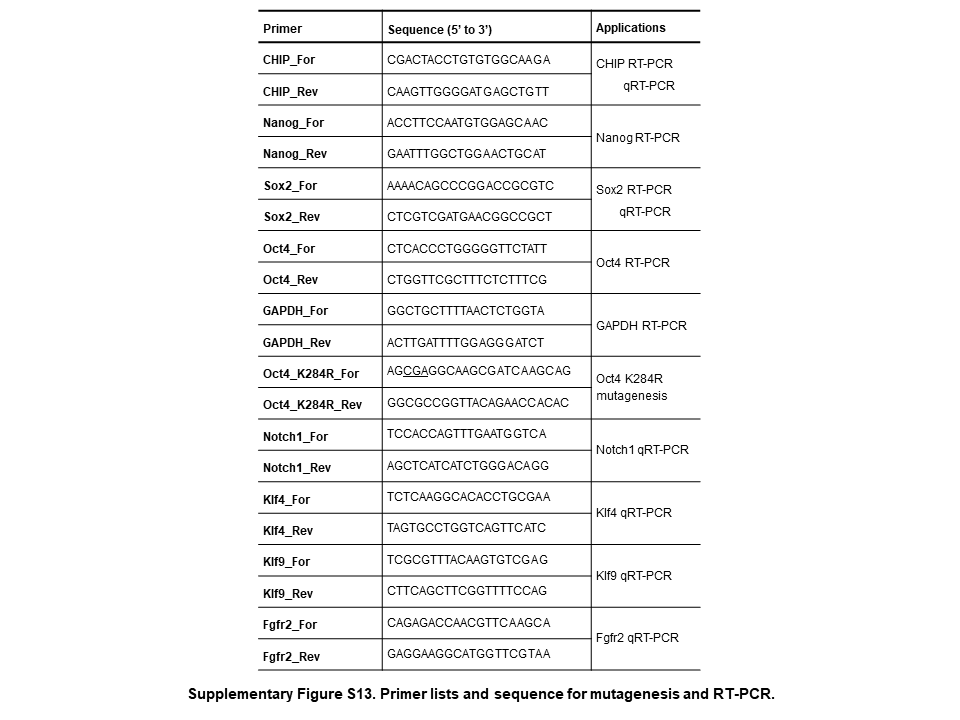

Supplement: Supplementary file 14 — supplementary figure S13 [file 41418_2018_79_MOESM14_ESM.tif]
